# Allografts for Medial Patellofemoral Ligament (MPFL) Reconstruction in Adolescent Patients with Recurrent Patellofemoral Instability: A Systematic Review

**DOI:** 10.3390/children10050840

**Published:** 2023-05-06

**Authors:** Filippo Migliorini, Nicola Maffulli, Stefan Söllner, Mario Pasurka, Joshua Kubach, Andreas Bell, Marcel Betsch

**Affiliations:** 1 Department of Orthopaedic, Trauma, and Reconstructive Surgery, RWTH University Hospital, 52074 Aachen, Germany; 2 Department of Orthopaedic and Trauma Surgery, Eifelklinik St. Brigida, 52152 Simmerath, Germany; 3 Department of Medicine, Surgery and Dentistry, University of Salerno, 84081 Baronissi, Italy; 4 School of Pharmacy and Bioengineering, Keele University Faculty of Medicine, Stoke-on-Trent ST4 7QB, UK; 5 Barts and The London School of Medicine and Dentistry, Centre for Sports and Exercise Medicine, Queen Mary University of London, Mile End Hospital, London E1 4DG, UK; 6 Department of Orthopaedic and Trauma Surgery, University Hospital of Erlangen, 91054 Erlangen, Germany

**Keywords:** patellofemoral instability, MPFL, allograft, adolescents, children

## Abstract

This systematic review updates the currently available evidence on medial patella-femoral ligament (MPFL) reconstruction using allografts. The outcomes were measured with patient-reported outcome measures (PROMs), redislocation and complication rates. This study was performed according to the 2020 PRISMA guidelines using the PubMed, Scopus, Web of Science databases, accessed in February 2023. Studies examining the clinical outcomes of MPFL reconstruction with allografts in adolescents and children with recurrent patellofemoral instability (PFI) were included. Data from three trials, including 113 surgical procedures in 121 children, were retrieved. 40% (48/121) of the included patients were girls. The mean age of the patients was 14.7 ± 0.8 years, and the mean follow-up length was 38.1 ± 16.5 months. With MPFL allograft reconstruction, the Kujala score improved by 14.7% (*p* < 0.0001) and the IKDC by 38.8% (*p* < 0.0001). The rate of dislocations was 5% (6 of 121), reoperation for instability was 11% (13 of 121), and subluxation was 2% (1 of 47). Conclusion: These results encourage the use of allografts for MPFL reconstruction in adolescent patients with recurrent patellofemoral instability. Though patellofemoral instability is common in clinical practice, the current literature lacks clinical evidence on allograft MPFL reconstruction. Additional high-quality investigations are required to properly establish the long-term advantages of allograft MPFL and its complication rate.

## 1. Introduction

Patients with patellar dislocation experience disabling symptoms, which limit their recreational activity levels and adversely affect their quality of life. Patella dislocations often occur with an annual incidence of 6 per 100,000 in the general population, but as high as 29 per 100,000 in the 10- to 17-year-old age group [1,2,3,4]. Twisting injuries of the knee or direct trauma to the medial aspect of the patella may result in a patella dislocation. Most patients with acute dislocation present a concomitant rupture of the medial patella-femoral ligament (MPFL) [5]. When the MPFL is ruptured, recurrent patellofemoral instability (PFI) may result. Obesity, trochlear dysplasia, patella alta or extensive knee valgus are also associated with a higher risk of recurrent PFI [6]. The MPFL runs from the undersurface of the vastus medialis obliquus to the medial aspect of the femur, functioning as the primary passive stabilizer of lateral patellar displacement between 0 to 30° of knee flexion [7]. In patellar dislocation, partial or complete rupture of the MPFL may occur [8]. Conservative treatment was previously considered the gold standard for MPFL injuries following patellar dislocations [9,10]. Recently, multiple proximal realignment procedures were introduced to treat PFI, which include MPFL reconstruction, lateral retinaculum release, MPFL repair or imbrication, as well as surgical procedures for distal realignment [11,12]. Reconstruction of the MPFL has become increasingly popular for the management of PFI, particularly in adolescent patients with open physes trying to avoid damage to the growth plate associated with bony procedures such as corrective osteotomies, tibial tubercle osteotomy or trochleoplasty [13,14,15]. MPFL reconstruction leads to satisfying clinical outcomes with low complication and redislocation rates [16,17].

Multiple surgical techniques have been described to reconstruct the MPFL using different graft types and fixation techniques [18,19,20]. However, the therapeutic algorithm for the management of patellofemoral instability is still debated [21]. Allografts for MPFL reconstruction have several advantages over autografts, including avoidance of donor-site morbidity and faster surgical time [22]. In adults, allografts are routinary used to reconstruct the MPFL. However, in children and adolescents, there is some hesitation to use allografts because of the suggested higher failure rates from longer incorporation rates and attenuation of the graft over time.

The current evidence on MPFL reconstruction with the use of allografts in adolescents is limited. Therefore, this systematic review was conducted to study the safety and efficacy of using allografts for MPFL reconstruction in adolescents with PFI by evaluating the midterm patient-reported outcome measures (PROMs) and complication rates. It was hypothesized that allograft MPFL reconstruction in adolescents is effective.

## 2. Materials and Methods

### 2.1. Eligibility Criteria

The inclusion criteria were: (1) all the clinical investigation reporting data on the outcomes of MPFL reconstruction using allografts in adolescent patients with recurrent patellofemoral instability; (2) studies with levels of evidence I to IV, according to the Oxford Centre of Evidence-Based Medicine [23]; (3) studies in English, Italian, German, Spanish and French; and (4) studies which clearly stated that allografts were used in all patients or reported data separately according to the type of graft used.

The exclusion criteria were: (1) studies which reported data on patients older than 18 years; (2) case reports, techniques, guidelines, editorials, comments, protocols, letters and reviews; (3) studies which performed autograft or synthetic MPFL reconstruction; (4) studies which included patients with congenital or acute patellofemoral instability; (5) studies which included patients with generalized ligament hyperlaxity; and (6) missing quantitative data on the endpoints of interest.

### 2.2. Search Strategy

This systematic review follows the preferred reporting items for systematic review and meta-analysis protocols (PRISMA) guidelines [24]. The PICO framework was used:

- Problem: recurrent patellofemoral instability;
- Population: skeletally immature patients;
- Intervention: allografts MPFL reconstruction;
- Outcomes: PROMs, return to sport, rate of complications;
- Follow-up: minimum 24 months follow-up.

The literature search was performed by two authors (F.M. & M.B.). In February 2023, Web of Science, Scopus, and PubMed were accessed using the following strategy: (patellofemoral OR patellar) AND (dislocation OR luxation OR instability) AND (medial patellofemoral ligament OR MPFL) AND (allograft) AND (surgery OR reconstruction) AND (skeletally immature OR children OR young OR adolescent OR open physeal). No filters and no time constraints were used for the databases search. The same authors screened the resulting titles and abstracts by hand. The full texts of potentially interesting articles were accessed. The bibliographies of the full texts were also screened by hand by the same authors. Of the authors’ disagreements, the final decision was taken by a third author (N.M.).

### 2.3. Data Extraction

Data extraction was performed by two investigators (F.M. and M.B.). Data were collected in Microsoft Office Excel (Microsoft Corporation, Redmond, WA, USA). Information on the author and the year of publication, journal of publication, level of evidence [23] and length of the follow-up were extracted. Data on patient demographic was also retrieved: mean age, number of females, number of patients and knees, and allograft source. Data on these PROMs were collected: the International Knee Document Committee (IKDC) [25] and the Kujala Anterior Knee Pain Scale [26]. The rate of further subluxation and/or dislocations and additional reoperations for persistent instability were retrieved.

### 2.4. Statistical Analysis

The main author (F.M.) performed the statistical analyses using the software IBM SPSS (version 25). For representative statistics, mean and standard deviation were used. Mean difference (MD), standard error (SE), *t*-value and 95% confidence of interval (CI) were used to assess the improvement of PROMs from baseline to the last follow-up. The paired two-tailed t-test was performed. Values of *p* < 0.05 were considered statistically significant.

## 3. Results

### 3.1. Search Result

**Figure 1:**
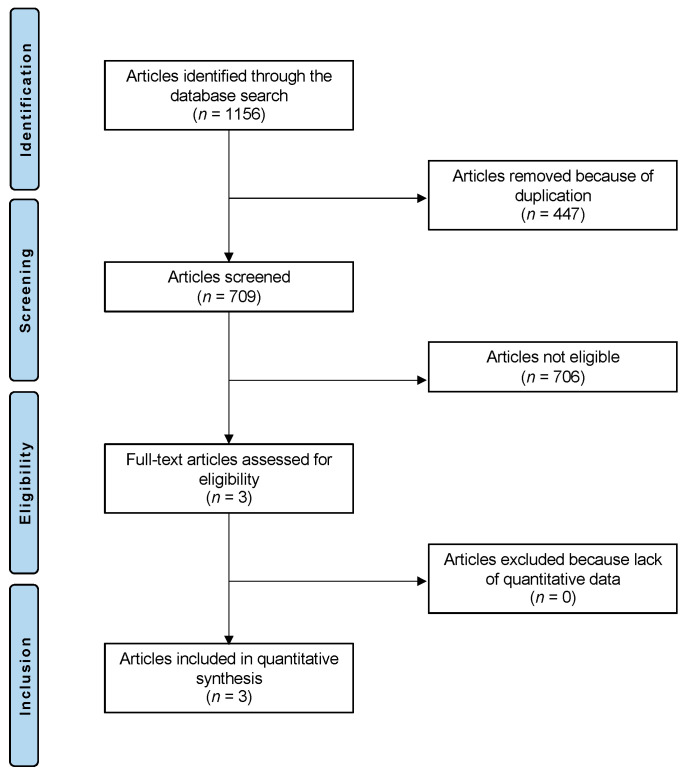
PRISMA flow chart of the literature search.

A total of 1156 articles resulted from the literature search. Of them, 447 were excluded as they were duplicates. An additional 706 full-texts were excluded as they did not match the eligibility criteria: not considering young and adolescents or not reporting on MPFL reconstruction (*n* = 281), not using allografts (*n* = 274), type of study (*n* = 135), focusing on acute or habitual patellofemoral dislocations (*n* = 12), language restrictions (*n* = 3) or focusing on patients with hyperlaxity (*n* = 1). Finally, three articles were included in the present investigation (Figure 1).

### 3.2. Demographic Data and Surgical Procedures

A total of 133 procedures (121 children) were analyzed. 40% (48 of 121 patients) were girls. The patients have a mean age of 14.7 ± 0.8 years at the time of surgery. Patients were evaluated at a mean of 8.1 ± 16.5 months follow-up. Demographic data of the included studies are shown in Table 1.

Several allografts were used: semitendinosus (*n* = 46), gracilis (*n* = 55), peroneus longus (2), tibialis anterior (*n* = 1), fascia lata (*n* = 22), and unspecified (7). Two authors [28,29] combined MPFL reconstruction with other procedures: loose body removal (*n* = 24), chondral debridement (*n* = 5), articular surface drilling for osteochondritis dissecans (*n* = 1), partial lateral meniscectomy (*n* = 1), medial quadriceps tendon femoral ligament (MQTFL) reconstruction (*n* = 25), and hemiepiphysiodesis (*n* = 5). The types of allografts used and the associated procedures are reported in Table 2.

### 3.3. Clinical Outcomes

At the last follow-up, the Kujala score improved by 14.7% (*p* < 0.0001) and the IKDC of 38.8% (*p* < 0.0001). These results are shown in greater detail in Table 3.

### 3.4. Complications

The rate of dislocations was 5% (6 of 121), reoperation for instability was 11% (13 of 121), and subluxation was 2% (1 of 47).

## 4. Discussion

Graft choice plays a pivotal role in knee ligament reconstruction [30,31]. However, graft choice in MPFL reconstruction in adolescents has not yet received the same amount of attention as, for example, ACL reconstruction. This study shows that MPFL reconstruction with allografts in adolescent patients with PFI leads to significant improvements in clinical outcome scores and a 5% rate of patella re-dislocation, and a subluxation rate of 2%. However, this procedure is also associated with a high reoperation rate of 11% when allografts are used. We were able to include a total of 133 surgical procedures in 121 children, which is low given the overall high incidence rate of patella dislocations in adolescents. The included studies also have a low level of evidence (III to IV), which is important when interpreting the results of this review.

Over the last few years, there has been an increased scientific and clinical interest in MPFL reconstruction concerning the refinement of its technique and clinical outcomes [32]. In the current literature, MPFL reconstruction was associated with a significant improvement in PROMs, a low rate of progression to recurrent PFI and complications [12,33,34]. However, when other anatomical factors in PFI are also present, such as an increased tibial tuberosity to trochlear groove distance, lower limb malalignment or patellar/trochlear dysplasia, isolated MPFL reconstruction may not be sufficient [35].

In this review, the mean age of the included patients was 14.7 years, with 40% of the population being girls. These findings are in accordance with previous studies with similar cohorts studying MPFL reconstruction [15,36,37]. Surprisingly, the majority of the included patients were males (60%), which is different to other studies in particular since young girls between 10 to 17 years have the highest risk for patellar dislocation [15,36].

Potential benefits of allograft MPFL reconstruction are less donor site morbidity and shorter surgical duration, which may result in less postoperative pain, lower graft site infection rates, less tenderness over the harvest site and shorter rehabilitation [38]. Furthermore, there are reports of decreased hamstring strength after graft harvest, as well as an increased risk of hamstring muscle strain following hamstring graft harvest [39]. Disadvantages, on the other hand, include the risk of disease transmission, lesser mechanical properties of the allograft, and higher costs. To overcome the disadvantages of allografts, synthetic grafts have also been advocated [32,40,41,42,43,44,45]. In a previous systematic review of 199 adults with recurrent PFI, synthetic graft MPFL reconstruction evidenced a significant improvement in PROMs at midterm follow-up [46]. The rate of re-dislocations and revisions was 2.5% and 1% [46], which was similar to this reported by previous reports on MPFL reconstruction with an autograft [33,47,48,49].

Some studies compared autograft versus allograft MPFL reconstruction [22,27,38,50,51,52,53,54]. In a recent meta-analysis of 474 adults with recurrent PFI, allograft MPFL reconstruction was associated with a significant increase in PROMs [55]. This improvement was similar to those achieved by patients who have undergone autograft MPFL reconstruction. A similarity was found in the rate of persistent instability sensation and revision [55]. However, patients who underwent allograft MPFL reconstruction evidenced a statistically significant lower rate of re-dislocations compared to those who underwent the autograft technique [55].

Although allograft reconstruction of the MPFL has been widely used in adults, the ideal graft type source and surgical technique have not yet been established in adults or in adolescents, given the lack of large comparative trials [56,57,58]. In a previous trial comparing MPFL reconstruction in adults with autograft versus allograft, no significant differences in terms of clinical outcomes between the graft types were found, while patients undergoing MPFL allograft reconstruction suffered from a higher patellar redislocation rate [59]. A trial by Flanigan, et al., in 87 patients comparing allograft versus autograft MPFL reconstruction showed low recurrent patellar dislocation rates (<3%) for both graft types [38], concluding that at least in this adult population, both graft types can be used without expecting significant differences in clinical outcomes. A systematic review including 1065 MPFL reconstructions revealed no statistically significant difference in terms of recurrent instability using allografts compared to autografts, while autograft MPFL reconstruction led to greater improvements in Kujala scores [60]. A previous systematic review and meta-analysis of the same study group on adult MPFL reconstruction demonstrated that allografts could be a feasible alternative to autografts, with similar PROMs, revision rates and persistent instability [55].

In skeletally immature patients, the femoral insertion of the MPFL is closed to the growth plates. The relationship between the Schöttle point and the growth plate is still debated. Indeed, the average proximity of the Schöttle point to the growth plate is approximately between 3.2 and 8.5 mm, with females having a lower distance compared to males [61,62,63]. During femoral MPFL fixation, the surgeon must consider the sizes of the drill and the anchors or interference screws to avoid growth plate damage. For the same reason, the angle of inclination of the drill should be controlled under fluoroscopic control. Hardware-free MPFL reconstruction could represent an option in skeletally immature patients [64,65,66,67,68,69,70,71]. In a previous systematic review, hardware-free MPFL reconstruction demonstrated midterm clinical improvement with a low rate of redislocation [72].

In skeletally immature patients, the lower limb alignment should not be overlooked. Genu valgum is an important pathoanatomical predisposing risk factor for patellofemoral instability [73]. In skeletally immature patients with genu valgum, hemiepiphysiodesis could be recommended in combination with MPFL reconstruction. Values of genu valgum greater than 10 degrees could represent a risk factor for patellofemoral instability [74,75]. A previous study on children with patellofemoral instability who underwent hemiepiphysiodesis for recurrent dislocation demonstrated an improvement in symptoms in 90% of the patients [76]. Among the included studies, Spang, et al., [29] also combined hemiepiphysiodesis in children with genu valgum. No patients who underwent guided growth developed recurrent instability at follow-up [29].

Graft cost and allograft availability need to be discussed when comparing allograft versus autograft MPFL reconstruction [22]. So far, no studies have directly compared the associated cost for MPFL reconstruction in adolescent patients; however, multiple studies exist of this type for adult anterior cruciate ligament reconstruction. Summarizing these studies, anterior cruciate ligament reconstruction using allografts is more expensive than autograft reconstructions, which is not balanced out by a shorter surgical duration [77,78].

While discussing the findings of this systematic review, it must be noted that there is some heterogeneity in terms of allograft types (semitendinosus, gracilis, peroneus longus, tibialis anterior, fascia lata) used in the included studies. Previous investigations have compared the influence of different graft types on MPFL reconstruction in the adult population. In 2016 Stephen, et al., performed cadaveric MPFL reconstructions using different graft types: gracilis tendon, quadriceps tendon, and tensor fasciae latae allograft [79]. Femoral tunnel placement and graft tensioning are more important for restoring the patella-femoral kinematics than the graft selection for MPFL reconstruction in cadavers. This has also been confirmed both in clinical trials with good short- and midterm outcomes and in a systematic review demonstrating no clinical differences for patients who have undergone MPFL reconstruction with different graft types [79,80,81].

In the present study, a higher percentage of females has been evidenced. This is in contrast with previous evidence, which stated that patellofemoral instability is more common in females, given their increased incidence of generalized ligamentous laxity and anterior knee laxity [82,83].

This systematic review has several limitations. For example, the small number of 133 procedures from three studies that were included in this study and the low level of evidence are limiting factors. There was also a high level of heterogeneity between the studies in terms of graft types and surgical techniques, which is considered a source of bias: given the lack of available data, no subgroup analysis was possible. The mean follow-up of the included studies was 38.1 months, allowing only short to midterm conclusions about the efficacy and safety of allograft for MPFL reconstruction in adolescents. Although patellar instability in adolescents is a frequent clinical problem, there is a lack of large prospective long-term clinical trials. Additional studies should be performed to overcome the current limitations, with trials including more homogeneous indications and characteristics and more information about patient-specific risk factors. In addition, it must be noted that two of the three studies used PROMs that have not been validated in the pediatric population, with Spang et al. being the only group to use the Pedi-IKDC [29].

Although the juvenile onset of patellofemoral instability, evidence on surgical alignments in skeletally immature patients with patellofemoral instability is lacking. Given the avoidance of the harvesting site, allograft MPFL reconstruction could be an interesting option in this population. However, evidence is lacking, and additional investigations are required to validate the results of the present study on a larger scale.

## 5. Conclusions

These results encourage the use of allografts for MPFL reconstruction in adolescent patients with recurrent patellofemoral instability. To establish the proper advantages of allografts, long-term comparative investigations are required.

## Figures and Tables

**Table 1:** Patient baseline demographic (LoE: level of evidence).

Author and Year	Journal	LoE	Mean Age	Follow-Up (*Months*)	Girls (*n*)	Patients (*n*)	Procedures (*n*)
Matuszewski, et al., 2018 [27]	Arthroscopy	III	15.00	24.00	10.00	22.00	22.00
Quinlan, et al., 2022 [28]	Arthroscopy, Sports Med, Rehab	III	15.40	55.20	19.00	58.00	67.00
			13.50	49.20	9.00	16.00	17.00
Spang, et al., 2019 [29]	J Ped Orthop	IV	15.00	24.00	10.00	25.00	27.00

**Table 2:** Types of allografts and associated procedures (OCD: osteochondritis dissecans; MQTFL: medial quadriceps tendon femoral ligament).

Author and Year	Type of Allograft (*n*)	Associated Procedures (*n*)
Matuszewski, et al., 2018 [27]	fascia lata (22)	none
Quinlan, et al., 2022 [28]	Semitendinosus (35), gracilis (25), peroneus longus (1), tibialis anterior (1), unspecified (5)	Loose body removal (21), chondral debridement (4), OCD drilling (1)
	Semitendinosus (11), gracilis (3), peroneus longus (1), unspecified (2)	Loose body removal (3), chondral debridement (1), partial lateral meniscectomy (1)
Spang, et al., 2019 [29]	Gracilis (27)	MQTFL reconstruction (25), hemiepiphysiodesis (5)

**Table 3:** Results of PROMs (FU: follow-up; MD: mean difference; SE: standard error; CI: confidence interval).

PROM	At Baseline	At Last FU	MD	SE	95% CI	T	*p*
Kujala Score	75.5 ± 2.2	90.2 ± 6.1	14.7	0.648	13.4 to 15.9	22.7	<0.0001
IKDC	38.9 ± 0.4	77.6 ± 0.4	38.7	0.1	38.5 to 38.8	684.1	<0.0001

## Data Availability

Data is contained within the article.
